# Supplementary material for: Interactions of Chemically Synthesized Ferrihydrite Nanoparticles with Human Serum Transferrin: Insights from Fluorescence Spectroscopic Studies
Source: Int J Mol Sci. 2021 Jun 29;22(13):7034. doi: 10.3390/ijms22137034 (PMC8268179; doi:10.3390/ijms22137034)
Supplement: Supplementary file 1 [file ijms-22-07034-s001.zip › ijms-1261687-supplementary.pdf]

Supplementary Material

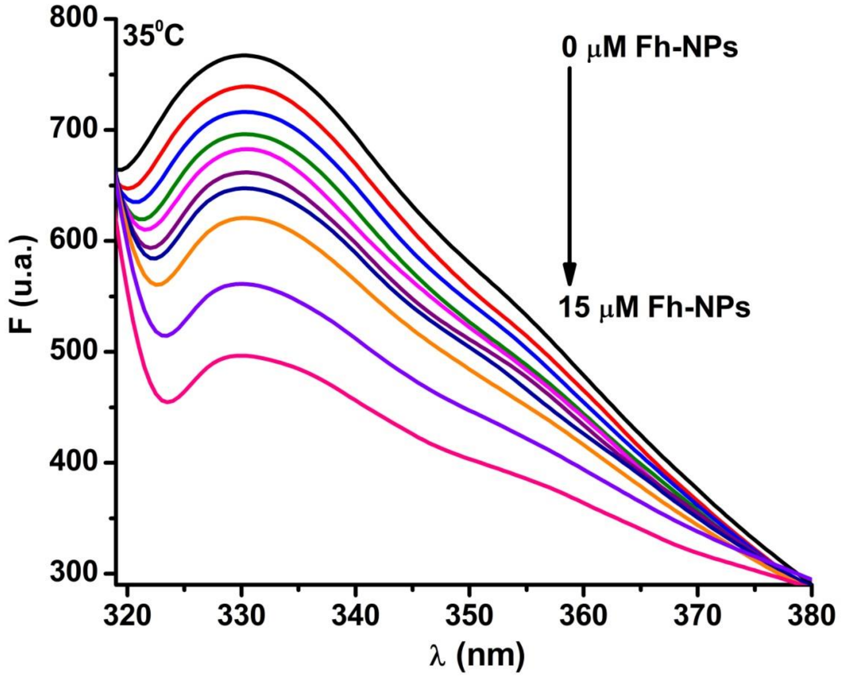

Figure S1: The fluorescence emission spectra of HST (3 μM) in the presence of Fh-NPs (0–15 μM) at 35 °C.

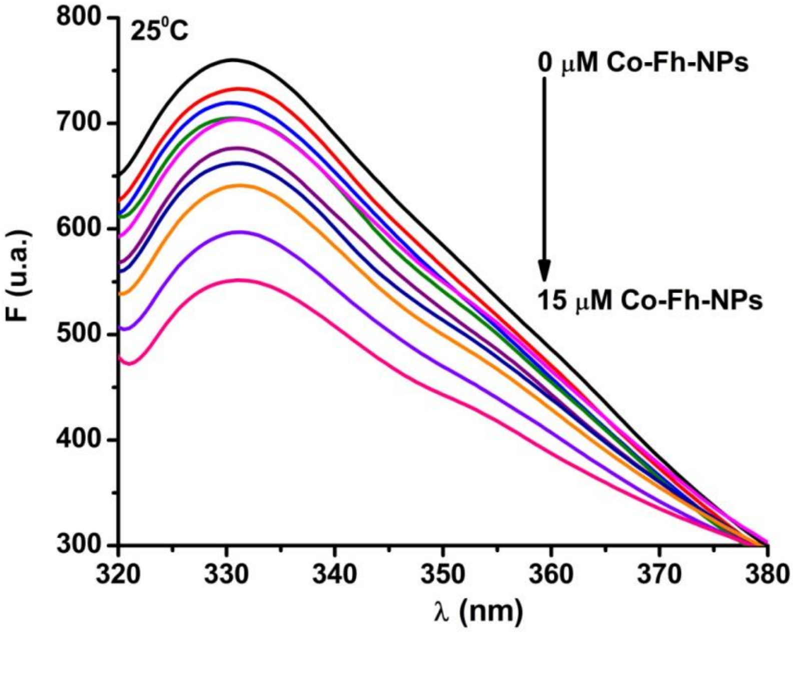

Figure S2: The fluorescence emission spectra of HST (3 μM) in the presence of Co-Fh-NPs (0–15 μM) at 25 °C.

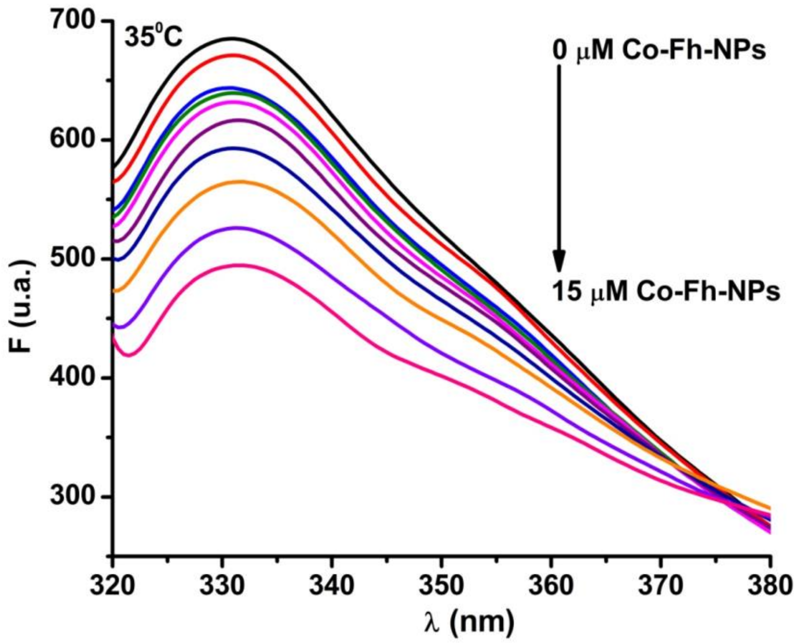

Figure S3: The fluorescence emission spectra of HST (3 μM) in the presence of Co-Fh-NPs (0–15 μM) at 35 °C.

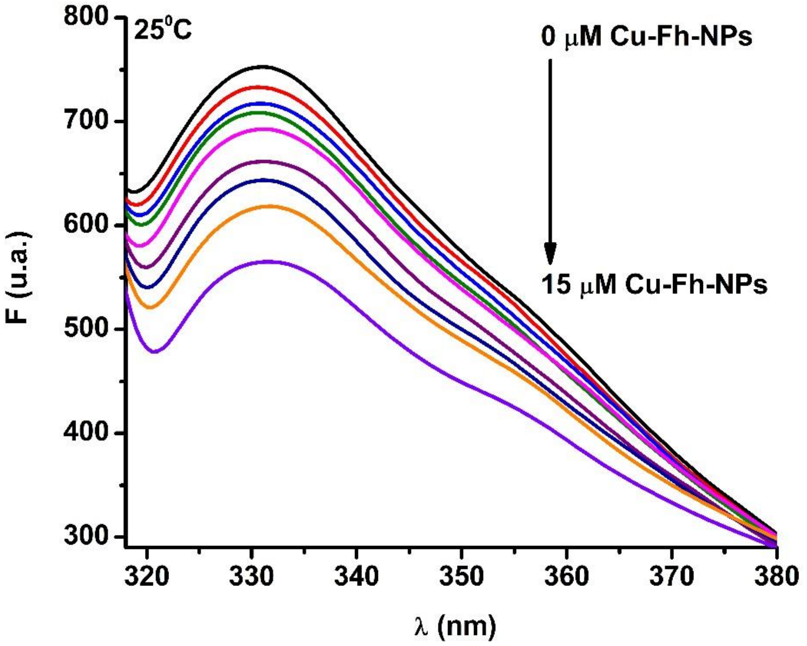

Figure S4: The fluorescence emission spectra of HST (3 μM) in the presence of Cu-Fh-NPs (0–15 μM) at 25 °C.

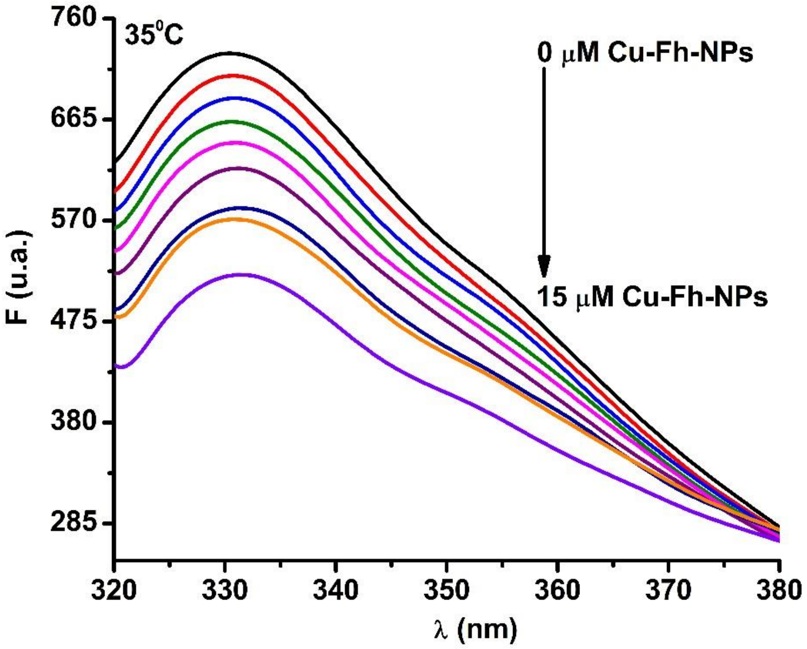

Figure S5: The fluorescence emission spectra of HST (3 μM) in the presence of Cu-Fh-NPs (0–15 μM) at 35 °C.

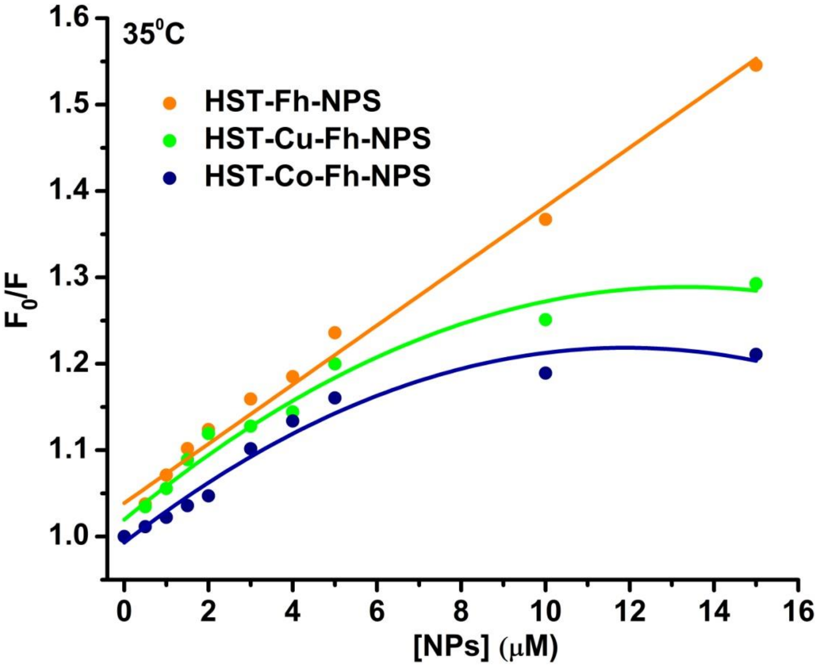

Figure S6: The Stern-Volmer representation of F_0/F vs. [NPs] for HST in complex with Fh-NPs (orange), Cu-Fh-NPs (green), and Co-Fh-NPs (blue) (0–15 μM) recorded at 35 °C.

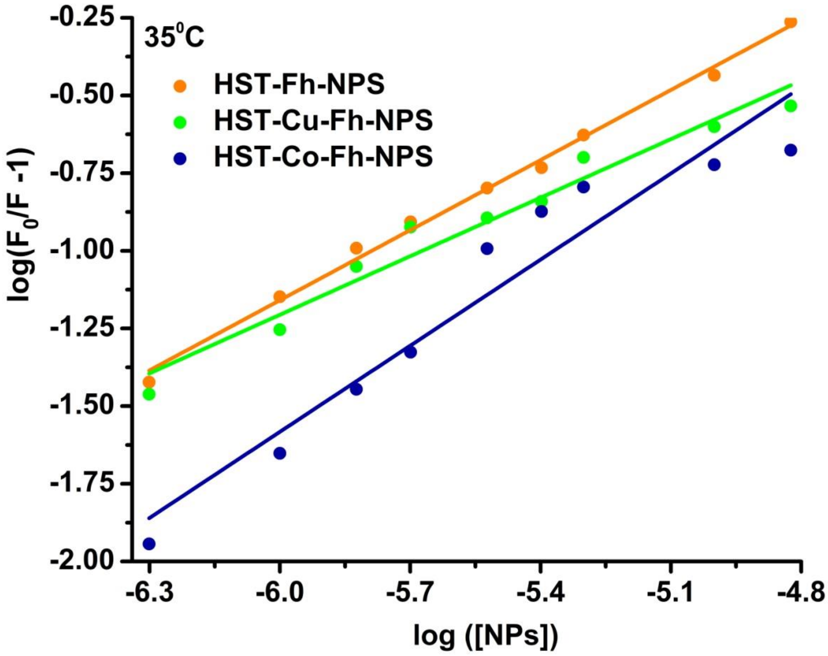

Figure S7: The Scatchard plot of log (F_0/F - 1) against log[NPs] for HST in complex with Fh-NPs (orange), Cu-Fh-NPs (green), and Co-Fh-NPs (blue) (0–15 μM) recorded at 35 °C.
